# Identification of Traumatic Bone Marrow Oedema: The Pearls and Pitfalls of Dual-Energy CT (DECT)

**DOI:** 10.3390/tomography7030037

**Published:** 2021-09-03

**Authors:** Giovanni Foti, Gerardo Serra, Venanzio Iacono, Claudio Zorzi

**Affiliations:** 1 Department of Radiology, IRCCS Sacro Cuore Don Calabria Hospital, 37024 Negrar, Italy; 2 Department of Anesthesia and Analgesic Therapy, IRCCS Sacro Cuore Don Calabria Hospital, 37024 Negrar, Italy; Gerardo.Serra@sacrocuore.it; 3 Department of Orthopaedic Surgery, IRCCS Sacro Cuore Don Calabria Hospital, 37024 Negrar, Italy; venanzio.iacono@Sacrocuore.it (V.I.); claudio.zorzi@sacrocuore.it (C.Z.)

**Keywords:** computed tomography, dual energy, bone marrow edema, trauma

## Abstract

Dual-energy computed tomography (DECT) has been reported to successfully identify bone marrow oedema (BME) in various traumatic settings. DECT has multiple strengths, including the availability of both a 3D view of the anatomical area studied and of high-resolution dual energy specific maps super-imposed onto conventional grayscale morphological images. Windowing can be used to enhance the visualization of BME by increasing the level of the super-imposed images. Conversely, by decreasing the level of the super-imposition of color-coded images, it is possible to progressively enhance the visualization of fine anatomical details, which is useful for diagnosing associated imaging findings. Importantly, bone sclerosis may represent an important pitfall for DECT, potentially generating both false positive and false negative findings by locally altering CT numbers. The aim of this paper was to evaluate the strengths and limitations of DECT in accurately detecting traumatic BME, by considering practical approaches to imaging at several anatomical sites.

## 1. Introduction

Bone marrow oedema (BME) represents a common clinical problem amongst the adult population [1]. BME is typically a response to an injury such as a fracture or conditions such as osteoarthritis. It occurs when fluid builds up in the bone marrow. In acute, post-traumatic settings, BME can be key for the identification of non-displaced fractures that are otherwise potentially missed by standard radiographs [2]. BME is often present following trauma, even in the absence of fractures [3], and subchondral BME, in particular, may prove a negative prognostic indicator for clinical outcomes [4,5]. Furthermore, additional imaging findings, such as ligamentous injuries, cartilage loss, or osteochondral lesions (OCL), may assist in diagnosis and prognostic assessment [3,6,7,8].

Generally, magnetic resonance imaging (MRI) is the preferred method for diagnosing BME [9,10]. However, MRI cannot be routinely used for all patients, owing to contraindications and its limited availability, especially in acute or traumatic settings. Computed tomography (CT) is the most frequently used imaging tool for body and skeletal trauma, and dual-energy computed tomography (DECT), in particular, has previously successfully identified BME in the spine [11] and in the lower and upper limbs [12,13].

In a traumatic setting, DECT has several strengths, such as a large gantry and rapid scanning time, in which virtually no motion artifacts are produced. In addition, claustrophobia, a common obstacle to MRI scanning, is not an issue. Furthermore, while DECT images can be used to identify BME, standard CT images can help in identifying subtle morphological changes that could prove key for diagnosis, thus increasing the reliability and reading confidence amongst less experienced readers, and reducing the overall reading time in general [14]. A further benefit is the possible detection of imaging findings associated with BME, such as OCL or hip avascular necrosis [3,7,8].

In recent metanalyses, DECT achieved high diagnostic accuracy values for the detection of BME (sensitivity—85%; specificity—97%). Importantly, DECT showed excellent diagnostic performance for both the spine/appendicular skeleton (sensitivity—84%/84%; specificity—98%/93%) [12,13].

This paper will describe the role of DECT in the assessment of post-traumatic BME, highlighting specific problems regarding the acquisition and interpretation of DECT images that could prove relevant to clinical practice.

## 2. Imaging Protocol

A second and a third generation dual-source CT scanner (Somatom Definition Flash and Force, Siemens Healthcare, Forchheim, Germany) are both routinely used at our institution. Tube voltages are set at 80/140 kV and 80/150 kV, in each scanner, respectively, using a tin filter. The predefined tube current–time product is set at a ratio of 1.6:1 (tube A, 220 mAs; tube B, 138 quality reference mAs) for imaging of the ankle, knee, wrist, and elbow, with detector collimation set at 32 × 0.6 mm, pitch at 0.6, and automated attenuation-based tube current modulation in use (CARE dose 4D; Siemens Healthcare). An increase of tube-current time-product values up to 300 and 228 mAs is often required to successfully scan the hip and shoulder, especially in cases of greater anatomical thickness, so as to reduce artifacts arising from photon starvation and scattering. The scanned regions are positioned in the center of the DE FOV, in order to avoid the artifacts typically seen at the periphery of the DE area.

## 3. Imaging Interpretation

By using the well-established three-material decomposition technique, virtual non-calcium images can be reconstructed from soft-tissue kernel (Qr32) images, yielding a better signal-to-noise ratio with respect to bone window images.

Standard 120 kVp 1 mm reconstructed images are evaluated using bone and soft tissue windows stored in the PACS archiving system, and DECT 3D and 2D images are evaluated on a dedicated offline workstation (SyngoVia^®^ VB20; Siemens, Erlangen, Germany). This allows for adequate windowing and post-processing of images. The most relevant DECT images can then be archived in PACS, as necessary.

**Figure 1:**
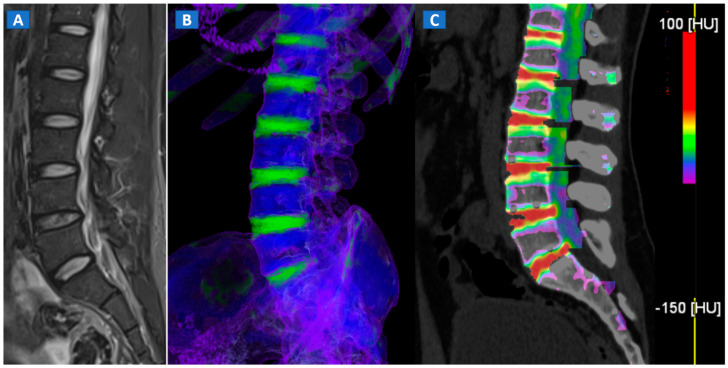
A 43-year-old male with low back pain following high-energy trauma, with no vertebral fractures. On the sagittal STIR MRI image (**A**), all lumbar vertebral bodies show a normal shape and signal intensity. On the 3D DECT image, on the para-sagittal plane (**B**), normal vertebral bodies are coded in blue, whereas density increases, due to increased water content, are coded in green. As expected, the normal intervertebral discs are coded in green. In addition, normal cortical bone is also coded in green, due to an incomplete subtraction process. On the corresponding 1 mm sagittal 2D DECT image (**C**) there is a color lookup table that codes bone marrow and areas of oedema in shades of green-yellow to orange-red. DE-specific information has been fused with conventional grayscale morphological images (thickness, 1 mm; increment, 1 mm). The range of densities is set between −150 and 100 HU, with underlying normal bone visualized without any superimposition for density values below the −50 HU cut-off.

**Figure 2:**
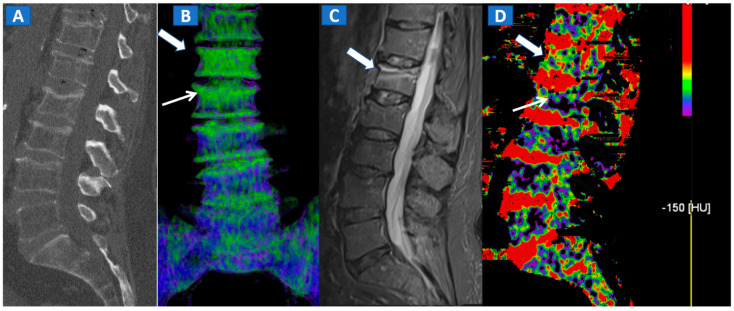
A 62-year-old female with traumatic low back pain following a fall. On the sagittal standard 1 mm CT image (**A**) it is not possible to identify the fresh fracture. On the coronal 3D DECT image (**B**), BME of the L1 vertebral body is coded in green (thick arrow), with some mild oedema depicted on the L2 superior endplate (thin arrow). On the sagittal STIR MRI image (**C**), a fresh fracture of the L1 body is confirmed, with mild oedema located close to the superior endplate (thick arrow). There is no BME of the L2 body apparent on the STIR MRI image. On the corresponding 2D DECT image (**D**), the maximum level of superimposition of color-coded maps was used to confirm the presence of mild oedema on the L1 vertebral body (thick arrow) and to rule out the presence of significant oedema on the L2 body (thin arrow), thus avoiding a false positive finding.

DECT 3D images are usually evaluated first, coding BME in shades of green and coding normal bone in blue (Figure 1). These images serve to provide a clear visual overview of the whole anatomical area, prior to any focus on specific findings. 3D images are usually more sensitive for depicting BME than 2D images (Figure 2). 2D images are analyzed by super-imposing the DE-specific information onto conventional grayscale morphological images (thickness, 1 mm; increment, 1 mm). Usually, a color lookup table is applied, which codes BME in shades ranging from green-yellow to orange-red (Figure 1C), with a range of densities set between −150 and 100 HU (Figure 1C). Superimposed color-coded maps are utilized only when density values are above the −50 HU cut-off. This approach can be useful in distinguishing between severe and mild BME (Figure 2). Additional windowing can also be carried out by increasing or decreasing the level of super-imposition of color-coded images in order to confirm or rule out subtler findings (Figure 2).

**Figure 3:**
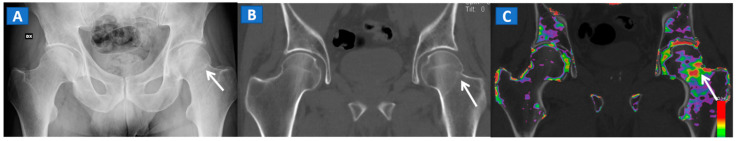
A 51-year-old male with a non-displaced left cervical fracture. On the coronal standard radiograph (**A**), a meshed fracture is suspected on the left side because of an apparent cortical irregularity (arrow). On the corresponding standard reconstructed 1 mm CT image (**B**), a subtle cortical irregularity is confirmed (arrow). On the corresponding DECT reconstructed coronal 1 mm image (**C**), severe BME clearly delineates the fracture line (arrow), which is surrounded by a mild oedema halo involving the trochanteric region.

However, in selected cases, for example in those of bone sclerosis, an alternative and widely used color-coding method, whereby spared bone is represented in violet and BME in shades of green, is preferred; this highlights an important pitfall of DECT [3,7,8]. Sclerosis is often identified on the acetabular roof, especially in patients suffering from degenerative osteoarthritis of the hip, or near to the pubis and sacroiliac joints, in the subchondral areas of knee and ankle, such as the tibial plateaus and talar dome, and adjacent to cortical bone in the upper extremities. Bone sclerosis can generate false positive findings, by locally increasing CT numbers (Figure 2), and can lead to false negative findings, due to excessive subtraction processes that can hinder BME detection. By slightly and progressively increasing the threshold for BME detection, however, it is sometimes possible to detect tiny areas of subchondral BME. In such cases, the parallel evaluation of standard CT images may help to delineate the distribution of sclerosis in expected areas, reducing the risk of misdiagnosis (Figure 3). Additionally, a comparison of the symptomatic and asymptomatic side can help confirm BME of the hip (Figure 3), or in cases where both an affected and unaffected joint are available in the FOV. In more difficult cases, the parameters may be adjusted initially to remove signs of BME from DECT images, before progressively increasing their visibility. By working on the BME threshold and on the level of super-imposition, it is usually possible to more accurately determine the location and distribution of BME.

Furthermore, DECT seems to be more accurate in diagnosing severe BME than milder, subtler oedema [8]. In selected cases, the quantitative assessment of DECT numbers, using an ROI in areas of less obvious BME, can help in achieving a differential diagnosis. However, thresholds may vary widely, depending on the anatomical area being evaluated, the age of the patient, and the imaging parameters used. Overall, qualitative assessment (sensitivity—85%; specificity—97%) is more accurate than quantitative assessment (sensitivity—84%; specificity—88%) [12,13].

## 4. The Spine

In cases of high-energy trauma, DECT images can help to rule out vertebral oedema, and reduce the reading time (Figure 1). When multiple vertebral fractures are present, DECT can identify fresh fractures that are typically characterized by the presence of BME (Figure 2). Furthermore, high resolution CT images can also be used to evaluate fine anatomical details such as transverse processes or posterior arches.

## 5. The Lower Limb: Hip, Knee, Ankle, and Foot

**Figure 4:**
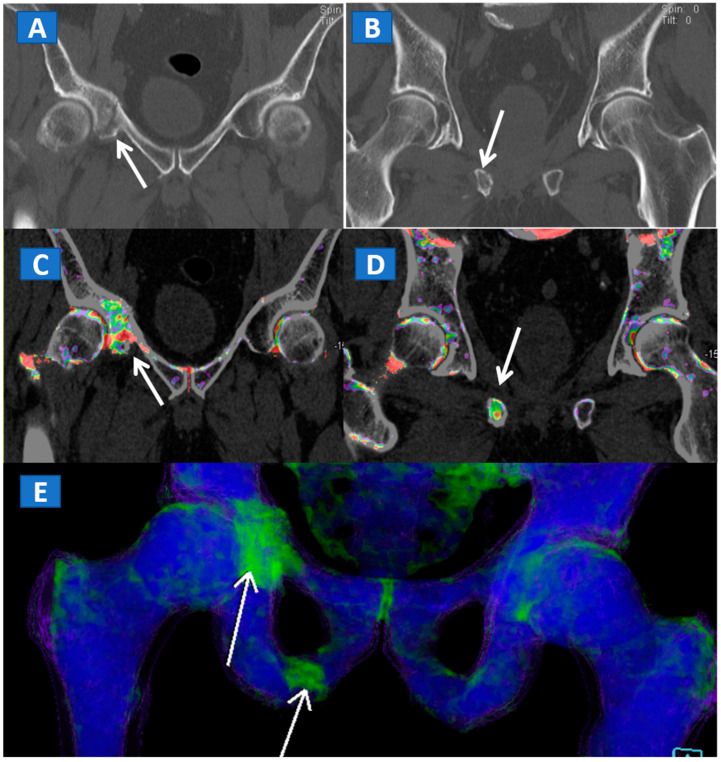
A 61-year-old female with non-displaced fractures of the right iliac and pubic bones. On the standard coronal 1 mm CT images (**A**,**B**) the iliac fracture is well depicted (arrow in (**A**)), whereas the pubic fractures could be easily missed (arrow in (**B**)). The presence of BME on the corresponding 1 mm coronal reconstructed DECT images (**C**,**D**) confirms the non-displaced fractures. The 3D coronal DECT map (**E**) yields a clear picture, depicting the fractures of the right side and ruling out the presence of BME on the left side.

DECT can also accurately depict BME in traumatic hip injuries [2]. When evaluating pelvic bones, multiple non-displaced but clinically relevant fractures frequently arise in clinical practice, for instance, untreated cervical hip fractures, which risk future displacement (Figure 3). In such cases, DECT can increase the diagnostic accuracy and reduce the reading time, allowing the radiologist to identify and focus on oedematous areas, which usually correspond to the facture line. DECT also helps in depicting multiple fractures (Figure 4), avoiding the risk of satisfaction of search errors.

**Figure 5:**
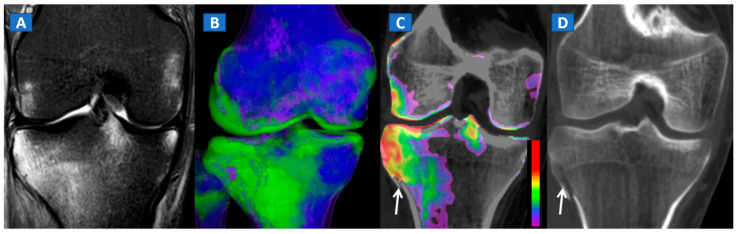
A 34-year-old male with a post-traumatic avulsion fracture of the lateral aspect of the proximal tibia. On the coronal STIR MRI image (**A**), multiple BME foci can be identified on the lateral aspect of the proximal tibia. On the coronal 3D DECT image (**B**), the distribution of BME foci is confirmed. On the corresponding 2D 1 mm reconstructed 2D DECT image (**C**), the BME appears more pronounced on the lateral aspect of the proximal tibia, adjacent to a cortical defect (arrow). The corresponding standard 1 mm reconstructed coronal CT image (**D**) confirms a minimally dislocated avulsion fracture (arrow).

**Figure 6:**
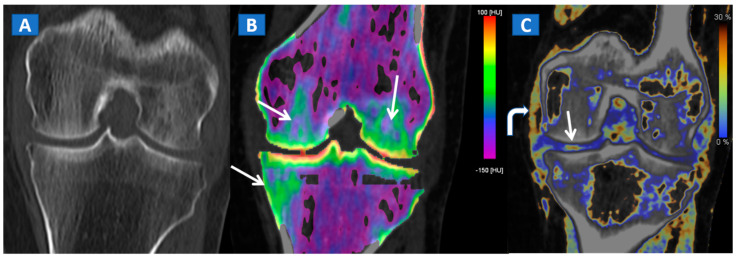
A 65-year-old female with a post-traumatic medial menisci rupture and diffuse BME. On the standard 1 mm reconstructed coronal CT image (**A**), there are no signs of fracture. On the corresponding 2D 1 mm reconstructed 2D DECT image (**B**), the BME appears more pronounced on the femoral condyles and on the medial tibial plateau (arrows). By using different color-coding (**C**), it was possible to identify a rupture of the medial menisci (straight arrow), with thickening and oedema of the medial collateral ligaments (curved arrow).

**Figure 7:**
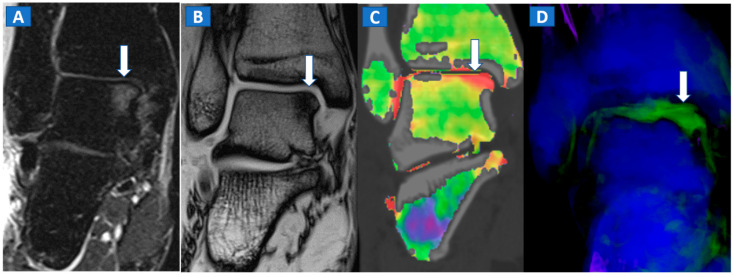
A 44-year-old male with post-traumatic OCL of the talar dome. On the coronal STIR MRI image (**A**), a hyperintense subchondral area of BME is depicted on the medial aspect of the talar dome (arrow), which is consistent with the diagnosis of OCL of the talus. The lesion appears hypointense on the T1-weighted images ((**B**); arrow). On the 1 mm reconstructed 2D coronal DECT image (**C**) and coronal 3D DECT map, the lesion appears as a subchondral oedematous area (arrow), due to its increased water content. On the corresponding 3D image (**D**) the BME is coded in green (arrow).

DECT can also accurately depict BME in post-traumatic fractures of the knee (Figure 5). Specifically, DECT can identify BME associated with ligamentous injuries following indirect trauma [6,15]. Moreover, additional color-coding can be used to better delineate meniscal tears (Figure 6). OCL, of the knee or ankle, are usually apparent from a markedly oedematous subchondral region (such as femoral condyle or astragalic dome), surrounded by a more subtle BME (Figure 7). With the availability of isotropic high-resolution CT images, it is possible to rule out migrated fragments and to confirm the integrity of the cortical bone.

Typically, the talus is the site most often involved in post-traumatic BME of the ankle. Subchondral bone sclerosis and thick cortical bone in the midfoot area may present significant limitations here [16].

## 6. The Upper Limb: Shoulder, Elbow, Wrist, and Hand

**Figure 8:**
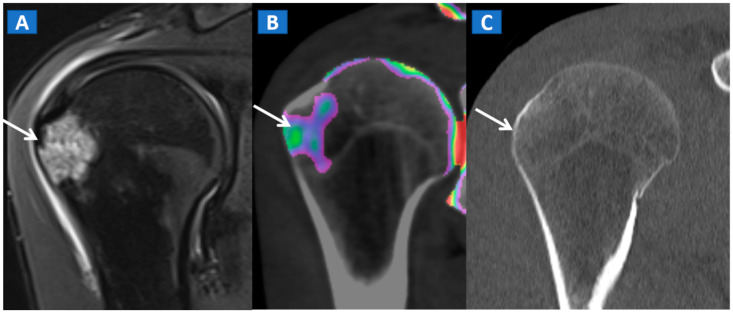
A 35-year-old male with traumatic BME of the humeral head. On the coronal STIR MRI image (**A**), post-traumatic BME of the humeral head is depicted as a hyperintense signal in the subcortical area (arrow). The presence of BME is confirmed on the 2D DECT coronal 1 mm reconstructed image (**B**) (arrow). The high resolution 1 mm coronal CT image with bone window (**C**), rules out the presence of cortical fractures.

In the post-traumatic shoulder, DECT can identify BME of the humeral head, even in the absence of cortical fractures (Figure 8). Furthermore, in cases of anterior dislocation, CT images can be used for diagnosing Hill-Sachs and bony Bankart lesions.

**Figure 9:**
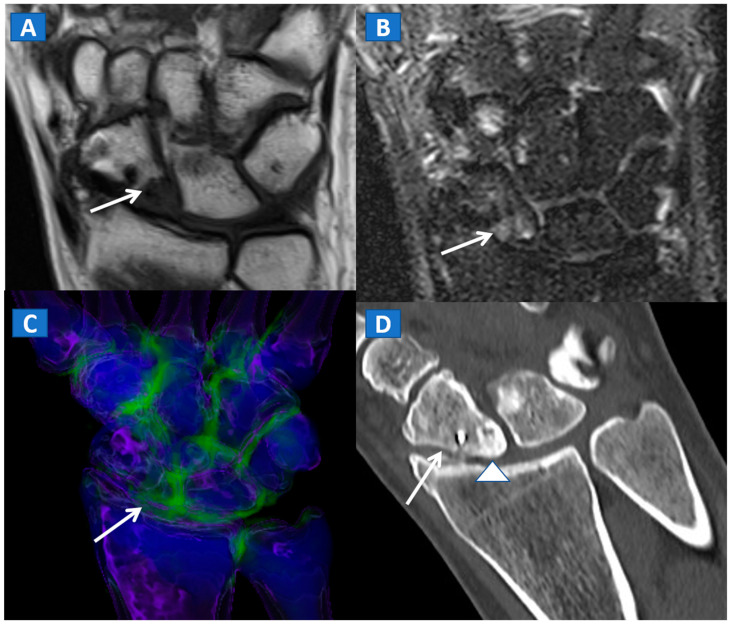
A 45-year-old male with persistent pain after surgical fixation of a middle-third carpal scaphoid fracture. On the coronal T1-weighted (**A**) and STIR (**B**) MRI images, metal-induced and motion artifacts impede the correct evaluation of the healing process. Furthermore, subtle BME is depicted proximally (arrow). On the 3D DECT image (**C**), some proximal BME can be confirmed (arrow). On the 1 mm reconstructed standard CT image on the coronal plane (**D**), the BME appears as an area of increased density (arrowhead). There are no signs of any residual fractures adjacent to the inserted metallic hardware.

In evaluating the elbow and wrist, the presence of BME as depicted by DECT, can assist the radiologist in identifying subtle meshed fractures. In addition, the ability of DECT to reduce metal artifacts may facilitate a more reliable evaluation of small anatomical structures, for example in cases of persistent pain after surgery, especially in patients where metallic hardware is present (Figure 9).

## 7. Conclusions

In our opinion, DECT represents a readily available, artifact-free imaging tool capable of identifying BME and associated bony and soft tissue imaging findings in traumatic settings.

## 8. Main Points

1. DECT is capable of identifying traumatic BME even in the absence of fractures.
2. DECT 3D images provide a clear visual overview of the whole anatomical area, and are quite sensitive in depicting BME.
3. Two-dimensional images are analyzed by super-imposing the DE-specific information onto conventional grayscale morphological images; windowing can be obtained by increasing or decreasing the level of super-imposition of color-coded images.
4. Bone sclerosis can generate both false positive and false negative findings, by locally altering CT numbers.
5. DECT cab be used for the detection of imaging findings associated with BME, such as OCL of the ankle and of the knee.

## Data Availability

All the data and cases were acquired at IRCCS Sacro Cuore Don Calabria Hospital, Negrar.
